# Characterization of a Romanian Pediatric Population with Eosinophilic Esophagitis

**DOI:** 10.3390/jcm13175041

**Published:** 2024-08-25

**Authors:** Iulia Florentina Tincu, Luiza Elena Bordei, Lucica Luminita Gales, Larisa Alexandra Duchi, Loredana Dobrescu, Bianca Teodora Chenescu

**Affiliations:** 1 Department of Paediatrics, “Carol Davila” University of Medicine and Pharmacy, 020021 Bucharest, Romania; iulia.tincu@umfcd.ro (I.F.T.);; 2 Gastroenterology Department, “Dr. Victor Gomoiu” Clinical Children Hospital, 022102 Bucharest, Romaniabiancaciurez@yahoo.com (B.T.C.)

**Keywords:** eosinophilic esophagitis, allergy, endoscopy

## Abstract

**Background/objectives:** An increase in the incidence of eosinophilic esophagitis (EoE) in children has been reported worldwide in the last decade. We conducted a study in a tertiary pediatric gastroenterology unit aimed at analyzing the clinical manifestations, biochemical markers, and endoscopic features of children with EoE in comparison to patients with non-eosinophilic esophagitis. **Methods**: This is a prospective analysis involving children with symptoms related to esophageal dysfunction, who had esophagogastroduodenoscopies with esophageal biopsies between January 2021 and April 2024 at “Dr. Victor Gomoiu” Clinical Children’s Hospital, in Bucharest, Romania. For the analysis, patients were considered in either Group 1, classified as EoE, or in Group 2, classified as reflux esophagitis. **Results**: Among the total of 72 patients diagnosed with esophagitis, 17 patients (Group 1—study group) were classified as EoE and 55 were classified as reflux esophagitis (Group 2—control group). The sex ratio analysis showed a male predominance in the study group (71% vs. 32%, *p* = 0.002). The main symptoms were regurgitation, eructation, and nausea. Dysphagia was present in two (11.76%) patients from Group 1. Eosinophilia was more prevalent in the EoE group than in the individuals with gastroesophageal reflux disease (GERD) (10 (58.85%) vs. 10 (18.18%, *p* = 0.001)), as well as the total IgE (11 (64.70%) vs. 6 (10.9%, *p* = 0.001)). **Conclusions:** Pediatric gastroenterologists need to be aware of EoE atypical presentation and perform adequate biopsies, mainly in children with refractory GERD-like symptoms, dysphagia, and food bolus impaction. Further analyses in terms of prognosis and treatment response should be addressed in longitudinal studies.

## 1. Introduction

Eosinophilic esophagitis (EoE) is a chronic disease affecting the esophagus and is characterized by an abnormal immune response. There is continuous infiltration and inflammation of the mucosa, mainly eosinophiles, leading to various symptoms that mimic esophagus dysfunction [1]. Both clinical and translational research in EoE is increasing worldwide, making it easier to understand the underlying mechanisms and treatment response. According to recent data, there is a growing number of both children and adults diagnosed with EoE [2], with the global pooled incidence and prevalence of EoE as high as 5.31 cases per 100,000 inhabitant-years (95% CI, 3.98–6.63; number of studies, 27; sample population, 42,191,506) and 40.04 cases per 100,000 inhabitant-years (95% CI, 31.10–48.98; number of studies, 20; sample population, 30,467,177), respectively [3]. Nevertheless, there is a greater adherence to invasive diagnostic protocols and a greater awareness of the medical information available about the disease [4]. The histopathological hallmark of EoE is eosinophilic infiltrations in esophageal biopsy specimens, with a minimum number of 15 eosinophils per high-power field (hpf) in the esophageal mucosa necessary for a positive diagnosis [5,6]. The pathogenesis of the disease is yet to be understood, but the latest information takes into consideration environmental factors, like diet and allergens [7]. Clinical aspects of EoE in children vary depending on age, from feeding refusal to food impaction [7]. The symptoms are often interchangeable with reflux-related clinical aspects like vomiting, food refusal, and abdominal pain [5,6]. Visualizing esophageal mucosa during upper gastrointestinal endoscopies in pediatric populations may increase the suspicion of eosinophilic infiltration in a wide range of lesions from stacked circular rings (“feline esophagus”), strictures (particularly proximal strictures), attenuation of the sub-epithelial vascular pattern, linear furrows to whitish papules (representing eosinophil micro-abscesses), and a small- caliber esophagus. In terms of treatment and follow-up procedures, there is a shift from the classical attitude based on symptoms and endoscopic remission to a target-to-treat strategy, involving prediction markers and fibrosis prevention.

Our department has experienced a growing need in the latest 4 years for endoscopic procedures in children, thus we were interested analyzing the symptoms, biochemical markers, and endoscopic features in children with EoE compared to patients presenting similar clinical data, without endoscopic and histologic characteristics of EoE.

## 2. Materials and Methods

This is a retrospective and prospective analysis involving children with symptoms related to esophageal dysfunction, who had esophagogastroduodenoscopies with esophageal biopsies between January 2021 and April 2024 at “Dr. Victor Gomoiu” Clinical Children’s Hospital, in Bucharest, Romania. This is a tertiary pediatric gastroenterology center, and the presented data are related to the patients addressed in this unit.

All clinical and laboratory data were collected from the hospital’s database. The inclusion criteria included a macroscopic aspect of esophagitis during an upper gastrointestinal endoscopy. Patients underwent a standardized upper gastrointestinal endoscopy, with an esophageal biopsy. The eosinophils were counted in terms of the hpf in the most densely infiltrated area. The diagnosis of EoE was considered when the sample included at least 15 eosinophils per hpf [5]. We excluded from the final analysis patients who had already been treated for EoE or who had other causes of esophageal eosinophilia (hypereosinophilic syndrome, infection, drug hypersensitivity, connective tissue disorder). The patients were divided into two working groups, taking into consideration the clinical data and histologic interpretation: the study group with EoE and the control group with non-EoE. Gastroesophageal reflux disease (GERD) was considered based upon the patient’s medical history, clinical symptoms, pH impedance studies, and upper contrast studies.

Data collected for both groups included information on three categories: (1) general information, namely age at diagnosis (years), gender (male/female), living area (rural/urban), Z score for body mass index at diagnosis, previously documented allergy diagnosis, respiratory or food allergies (yes/no answers), duration in months between first symptom and final diagnosis, and standard symptom questionnaire with yes/no answers for onset symptoms (abdominal pain, failure to thrive, dysphagia, halitosis, vomiting, lack of appetite, heartburn, nausea, weight loss, eructation, regurgitation, chest pain); (2) hematologic and biochemical characteristics, namely peripheral eosinophils, hemoglobin level, platelet count, C-reactive protein, total serum IgE, and 25 hydroxy vitamin D level; and (3) endoscopic and histologic analysis, namely the presence of longitudinal furrowing, decreased vascular pattern, whitish exudates, esophageal erosion, mucosal esophageal erythema, papules/plaques, trachealization, esophageal polyp, esophageal hernia, and eosinophil counts.

The statistical analysis was performed using IBM SPSS Statistics Version 22.0; mainly the chi-square test and Mann–Whitney U were used to compare the data between the 2 groups. A *p* value < 0.05 was considered statistically significant.

The study was approved by the Hospital Ethics Committee (no. 15444/22.09.2023) and all caregivers signed an informed consent form before adherence to the study protocol.

## 3. Results

Among the total of 72 patients diagnosed with esophagitis, 17 patients (Group 1—study group) were classified as EoE and 55 were classified as reflux esophagitis (Group 2—control group). The demographic characteristics, reported symptoms, and coexisting diseases of the patients in the analyzed groups are listed in Table 1. The period of time spent from the symptom onset until the final diagnosis was significantly longer in children with EoE than in individuals with gastroesophageal reflux (GERD) (*p* < 0.001).

The median age in Group 1 individuals (6.34 ± 1.34 years) was significantly higher than that of Group 2 individuals (4.44 ± 1.22 years). The sex ratio analysis showed a male predominance in the study group (71% vs. 32%, *p* = 0.002). There was a higher incidence of both food (35.29% vs. 21.81%, *p* = 0.002) and respiratory allergies (23.52% vs. 10.90%, *p* = 0.005) in the study group compared to the control group; the diagnosis was made by an allergologist based on a prick test and specific IgE, prior to the study. Previous atopic conditions (e.g., atopic dermatitis, asthma, allergic rhinitis, eczema, and IgE-mediated food allergy) were found in 10 (58.82%) patients from Group 1 and in 18 (32.72%) patients from Group 2 (*p* < 0.001).

The most frequent allergic conditions in Group 1 were allergic rhinitis (two patients), followed by food allergies (six patients), and asthma (two patients).

The timing interval spent between symptom appearance and diagnosis was longer in Group 1 (14.3 ± 2.1 vs. 6.7 ± 1.8 months, *p* < 0.005).

The main symptoms were abdominal pain, nausea, and eructation. Dysphagia was present in two (11.76%) patients from Group 1.

Eosinophilia was more prevalent in the EoE group than in the GERD individuals (10 (58.85%) vs. 10 (18.18%), *p* = 0.001), as well as an elevated total IgE (11 (64.70%) vs. 6 (10.9%), *p* = 0.001); no other biochemistry or hematological differences were detected (Table 2).

The pH/impedance (pH/MII) monitoring was performed in 32 (44.44%) of all patients, 8 (47.05%) in Group 1 and 24 (43.63%) in Group 2, considering that EoE and GERD can co-exist, according to the latest guidelines [5,6]. There was a high rate of pH/MII refusal by parents when the investigation was proposed for patients. Positive results for the 24 h esophageal pH/impedance monitoring were observed in two (40%) of the patients with EoE compared to 18 (75%) of the children from the non-EoE group (*p* = 0.005). More data on the test results are presented in Table 2.

The most frequent endoscopic lesions in the EoE group were longitudinal furrowing (*p* < 0.001), decreased vascular pattern (*p* < 0.001), and whitish exudates (*p* < 0.001) (Table 2). There was one patient with normal macroscopic mucosa and one patient with EoE presented with stenosis.

The median number of eosinophils/hpf was 66.67 ± 12.34 (range 19–120) in the EoE group and 6.2 ± 1.1 (range 0–12) in the GERD group.

The mean number of eosinophiles in the esophageal mucosa was 66.67 ± 12.34 in our patients. Other histologic features in the study were the superficial layering of the eosinophils, basal zone hyperplasia, eosinophilic micro-abscesses (clusters of >4 eosinophils), dilated intercellular spaces, surface epithelial alteration, and dyskeratotic epithelial cells.

## 4. Discussion

This study reports the first data of a Romanian child population diagnosed with EoE, which aimed to prospectively characterize this condition according to the ESPGHAN guidelines [2,8] in a single tertiary center. There were no data from previous publications that could be used to identify the overall incidence. Nevertheless, there are disparities among EoE incidence in Europe, varying from 1.5 to 10.9 (mean 5.1) per 100,000 children/year; there are higher rates of incidence in North America than in Europe, a variation reported according to the investigation period, regional distribution, and analysis methodology [2].

Our study finds a male predominance in the EoE patients, similar to the international literature [9,10], one of the explanations being that single nucleotide polymorphisms (SNPs) in the thymic stromal lymphopoietin (TSLP) gene and nonsynonymous SNPs in the TSLP receptor may be associated with EoE in male patients only [11].

There is an age variation regarding symptoms in patients with EoE. As reported before, clinical appearance in infants and toddlers is related to vomiting and feeding difficulties, while it manifests in children and adolescents mainly through abdominal pain, dysphagia, and food impaction [12]. In our study, the main symptoms were abdominal pain, lack of appetite, and regurgitation; dysphagia, although rare in our cohort, was the main distinguishing symptom between EoE patients and non-EoE patients [12]. The prevalence of failure to thrive was similar in the two groups analyzed; however, dysphagia, eructation during meals, and chest pain were more prevalent in Group 1. Mehta et al. did not report any nutritional deficiencies among EoE and GERD pediatric patients or any abnormalities in growth parameters [13]. Food impaction was not observed in our EoE group, although it is well-described in pediatric EoE individuals, sometimes with frequencies similar to adults [14]. There is an association between various conditions characterized by inflammation, like asthma, atopic dermatitis, and food allergies, and EoE [15]. In our cohort we identified a history of atopic disorders in 52.94% of patients, as seen in similar reports [10,16]. Also, peripheral eosinophilia was reported in 8.6–33% of patients [17], while our research showed that 58.85% of individuals had >400 eos/μL in peripheral blood. Patients having undergone a repair of their esophageal atresia in the neonatal period were found to have an increased risk of EoE, with microdeletion involving the Forkhead box transcription factor (FOXF1), as a common genetic abnormality [18]. None of our patients previously underwent an esophageal atresia surgical intervention. According to the latest publication from the European Society for Paediatric Gastroenterology, Hepatology and Nutrition (ESPGHAN), upper GI endoscopy with at least six biopsies from upper and lower levels of the esophagus (at least two samples from each level) remains the gold standard for EoE diagnosis and follow-up [8]. Specialists may use the EoE Endoscopic Reference Score, although further validation needs to be performed in the pediatric population. Most of the children (80%) in Group 1 were identified as having typical endoscopic features, similar to a recent report [19]. EoE is characterized by classic endoscopic findings, like linear furrows, concentric rings, whitish plaques, and strictures. Saeed et al. reported in his study that the majority (72%) of EoE patients had one of these findings [16], while Assiri et al. identified that linear furrows were the most common abnormalities [20]. In the meantime, a normal macroscopic esophagus in a patient with symptoms suggesting reflux should inspire gastroenterologists to perform biopsies, considering that various percentages of patients might have microscopic diagnostics that have been missed; Oliva et al. reported normal mucosa appearance in 17% of patients [12] and, in a Brazilian cohort, even larger percentages (45%) of EoE diagnostics with normal endoscopic mucosa were described [21]. In our study, there was only one patient (5.88%) without macroscopic findings, but with an esophageal eosinophilic infiltration of more than 15 eos/hpf.

There is no specific non-invasive biomarker for EoE. The number of eosinophiles in peripheral blood is more prevalent in EoE, but the association is not specific [22]. A total of 10 (58.85%) patients in our study had elevated peripheral eosinophilia and this was statistically significant compared to children in the control group. Considering the invasive character of patient surveillance, researchers have been searching for novel and more accessible criteria; in adult studies, the peripheral blood eosinophil count is demonstrated as a marker for the assessment of the treatment response [23], but in the child population there is no correlation in this area.

The gold standard for EoE diagnosis is considered to be biopsy findings with an increased intraepithelial esophageal eosinophil count, ≥15 eos/hpf [24].

There is little role for esophageal pH/MII in the diagnostic workup of EoE in children, although GERD could play a role in the pathogenesis of the disease; also, some studies have reported abnormal pH/MII concomitant with EoE [25]. We found a positive reflux pH/MII in two (40%) EoE patients.

Endoscopic treatment is reserved for complications, such as food impaction removal or dilation of esophageal strictures unresponsive to medical therapy. While in adults there are larger rates of dilatation, in the pediatric population there are lower rates and, in our study, the stenotic patient responded to steroid therapy.

Our study had limitations in terms of the small sample size, reflecting data from a single tertiary care hospital, and not everyone in the entire group presenting with GERD-like symptoms was involved in the pH/MII studies. There is a need for larger multicentric studies.

## 5. Conclusions

Pediatric gastroenterologists need to be aware of EoE atypical presentation and the need to perform adequate biopsies, mainly in children, with refractory GERD-like symptoms, dysphagia, and food bolus impaction. A high index of clinical suspicion is required mainly for infants and toddlers and proper invasive investigations, like endoscopy, should be used for proper diagnosis. There is a higher need to perform pH/MII studies in order to differentiate GERD patients. Further analyses in terms of the prognosis and treatment response should be addressed in longitudinal studies.

## Figures and Tables

**Table 1:** Baseline characteristics.

	Group 1 (EoE Patients)	Group 2 (Non-EoE Patients)	*p*-Value
Number of patients	17	55	
Age (mean ± SD, years)	6.34 ± 1.34	4.44 ± 1.22	0.005
Male (n, %)	12 (71)	17 (32)	0.002
Urban area (n, %)	16 (94.11)	35 (63.63)	NS
Z-score BMI (<−1) (n, %)	12 (71)	42 (73.36)	NS
Food allergy (n, %)	6 (35.29)	12 (21.81)	0.005
Respiratory allergy (n, %)	4 (23.52)	6 (10.90)	0.005
Time until diagnosis (mean ± SD, months)	14.3 ± 2.1	6.7 ± 1.8	0.005
Abdominal pain (n, %)	9 (52.94)	22 (40)	NS
Failure to thrive (n, %)	12 (71)	42 (73.36)	NS
Dysphagia (n, %)	2 (11.76)	0	NA
Halitosis (n, %)	2 (11.76)	15 (27.27)	<0.005
Vomiting (n, %)	8 (47.05)	28 (50.90)	NS
Lack of appetite (n, %)	6 (35.29)	22 (40)	NS
Heartburn (n, %)	3 (17.64)	10 (18.18)	NS
Nausea (n, %)	10 (58.82)	37 (67.27)	NS
Eructation (n, %)	14 (82.35)	38 (69.09)	<0.005
Regurgitation (n, %)	13 (76.47)	39 (70.90)	NS
Chest pain (n, %)	3 (17.64)	11 (20)	NS

Notes: n—number; SD—standard deviation; NA—not applicable; NS—non-significant; BMI—body mass index.

**Table 2:** Endoscopic, histologic, and biochemistry data.

	Group 1 (EoE Patients)	Group 2 (Non-EoE Patients)	*p*-Value
Normal mucosa (n, %)	1 (5.88)	0 (0)	NA
Longitudinal furrowing (n, %)	16 (94.11)	5 (9.09)	0.005
Decreased vascular pattern (n, %)	13 (76.47)	2 (3.63)	0.005
Whitish exudates (n, %)	14 (82.35)	1 (1.81)	0.005
Esophageal erosion (n, %)	15 (88.23)	42(73.36)	NS
Mucosal esophageal erythema (n, %)	16 (94.11)	48 (87.27)	NS
Papules/plaques (n, %)	11 (64.70)	2 (3.63)	0.005
Trachealization/rings (n, %)	9 (52.94)	0 (0)	NA
Esophageal polyp (n, %)	0 (0)	6	NA
Esophageal hernia (n, %)	0 (0)	0 (0)	NA
Mucosal eosinophil count (median ± SD)	66.67 ± 12.34	6.2 ± 1.1	0.005
Eosinophilia (>400 eos/μL) (n, %)	10 (58.85%)	10 (18.18%)	0.005
Hb (median ± SD) (g/dL)	12.7 ± 6.2	13.15 ± 7.6	NS
CRP (median ± SD) (mg/dL)	3.7 ± 1.2	2.2 ± 1.7	NS
PLT (median ± SD) (×10^3^/μL)	388.45 ± 66.67	289.72 ± 55.98	NS
Serum IgE (median ± SD) (IU/mL)	98.65 ± 12.33	44.24 ± 12.56	0.002
25 hydroxy vitamin D (median ± SD) (ng/mL)	32.38 ± 9.34	43.23 ± 13.32	NS

Notes: CRP—C-reactive protein; Hb—hemoglobin; PLT—platelet; NS—non-significant; NA—not applicable; n—number; SD—standard deviation; eos—eosinophils; IgE—immunoglobulin E; IU—international units.

## Data Availability

Data are contained within the article.
